# A Comprehensive Comparative Analysis of Sequence‐Based Deep Learning Models for Single‐Cell Genomics

**DOI:** 10.1111/cpr.70274

**Published:** 2026-08-23

**Authors:** Guoxia Wen, Jiaqi Li, Hanyu Wu, Jialing Fang, Yuting Fu, Mengmeng Jiang, Yuqing Mei, Rui Xu, Yuxuan Du, Siran Chu, Guoji Guo, Xiaoping Han, Jingjing Wang

**Affiliations:** ^1^ Bone Marrow Transplantation Center of the First Affiliated Hospital & Liangzhu Laboratory, Zhejiang University School of Medicine Hangzhou Zhejiang China; ^2^ Center for Stem Cell and Regenerative Medicine Zhejiang University School of Medicine Hangzhou Zhejiang China; ^3^ Zhejiang Provincial Key Lab for Tissue Engineering and Regenerative Medicine, Dr. Li Dak Sum & Yip Yio Chin Center for Stem Cell and Regenerative Medicine Hangzhou Zhejiang China; ^4^ Institute of Hematology Zhejiang University Hangzhou Zhejiang China

## Abstract

To streamline the application of sequence‐based DL methods in single‐cell genomics, we established a two‐layer CNN as our baseline model. We focus our benchmark on how data characteristics, hyperparameter optimization, and advanced model architectures impact performance across sequence‐to‐expression and sequence‐to‐regulation tasks. A key contribution of our study is the exploration of multi‐task learning (MTL) frameworks for mitigate technical sparsity. We demonstrated that MTL significantly enhances the modeling of cellular heterogeneity, evaluating the effectiveness of task grouping and balancing strategies, with particular focus on the prediction of rare cell types. Our comprehensive comparative analysis provided an actionable framework and valuable insights for guiding future research endeavors and facilitating the development of the sequence‐based DL models capable of superior predictive performance in single‐cell genomics.

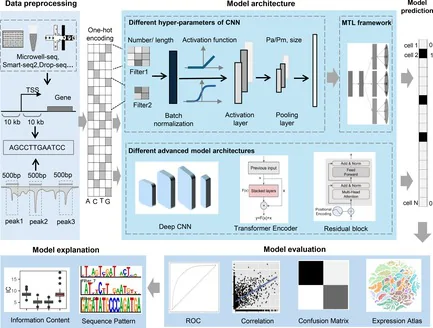

To the Editor,

1

Although single‐cell genomics has transformed our understanding of cellular heterogeneity, the inherent high‐dimensionality, sparsity, and technical noise of these data pose major challenges for conventional computational methods [1]. Sequence‐to‐function deep learning (DL) models have emerged as powerful tools for predicting gene expression and chromatin accessibility, helping decode the regulatory grammar [2, 3]. Convolutional Neural Networks (CNNs), in particular, are favoured for their efficiency and interpretability [4]. However, the rapid proliferation of models, data modalities, and analytical strategies has outpaced systematic evaluation, necessitating a benchmark of current methodological standards.

To streamline the application of sequence‐based DL methods in single‐cell genomics, we established a two‐layer CNN as our baseline model. This choice is directly informed by established architectures such as Nvwa [5], which identify this structure as the minimal effective architecture for sequence‐to‐function tasks in genomics. Specifically, the first layer (128 filters, kernel size 15) is designed for motif detection, capturing local regulatory elements equivalent to Position Weight Matrices (PWMs), while the second layer (256 filters, kernel size 3) serves to integrate these signals, capturing the spatial dependencies and complex regulatory syntax between elements. We focus our benchmark on how data characteristics, hyperparameter optimization, and advanced model architectures impact performance across sequence‐to‐expression and sequence‐to‐regulation tasks. A key contribution of our study is the exploration of multi‐task learning (MTL) frameworks for mitigating technical sparsity. We demonstrated that MTL significantly enhances the modelling of cellular heterogeneity, evaluating the effectiveness of task grouping and balancing strategies, with particular focus on the prediction of rare cell types. Our comprehensive comparative analysis provided an actionable framework and valuable insights for guiding future research endeavours and facilitating the development of the sequence‐based DL models capable of superior predictive performance in single‐cell genomics.

We initially evaluated the performance of our baseline CNN model across three biological systems: mESCs, human PBMCs, and human pancreas (Table S1). The baseline model performance varied substantially across the three datasets. For the classification task, the model achieved the highest predictive accuracy on the mESCs dataset with an average AUROC of 0.84, followed by the pancreas (average AUROC = 0.78) and the PBMCs (average AUROC = 0.73) dataset (Figure 1A; Figure S1A,B, and Table S2). A consistent performance trend was observed for the regression task (Figure S1C–E). To investigate the underlying drivers of these differences, we analysed the relationship between data density (indexed by the number of detected gene per platform) and model performance. We observed a strong positive correlation between gene detection numbers and PCC values across all three datasets. Specifically, the mESC dataset showed more gene detection numbers than PBMC and pancreas, yielded the highest overall performance. For instance, while Smart‐seq2 delivered the highest prediction performance in the mESCs dataset (AUROC = 0.86 and PCC = 0.63) with a mean of 8741 detected genes, its performance dropped to intermediate performance in the other two datasets (Figure 1A; Figure S1A–E) corresponding to a significant decrease in mean detected genes of 2606 and 5848, respectively (Table S2).

**FIGURE 1:**
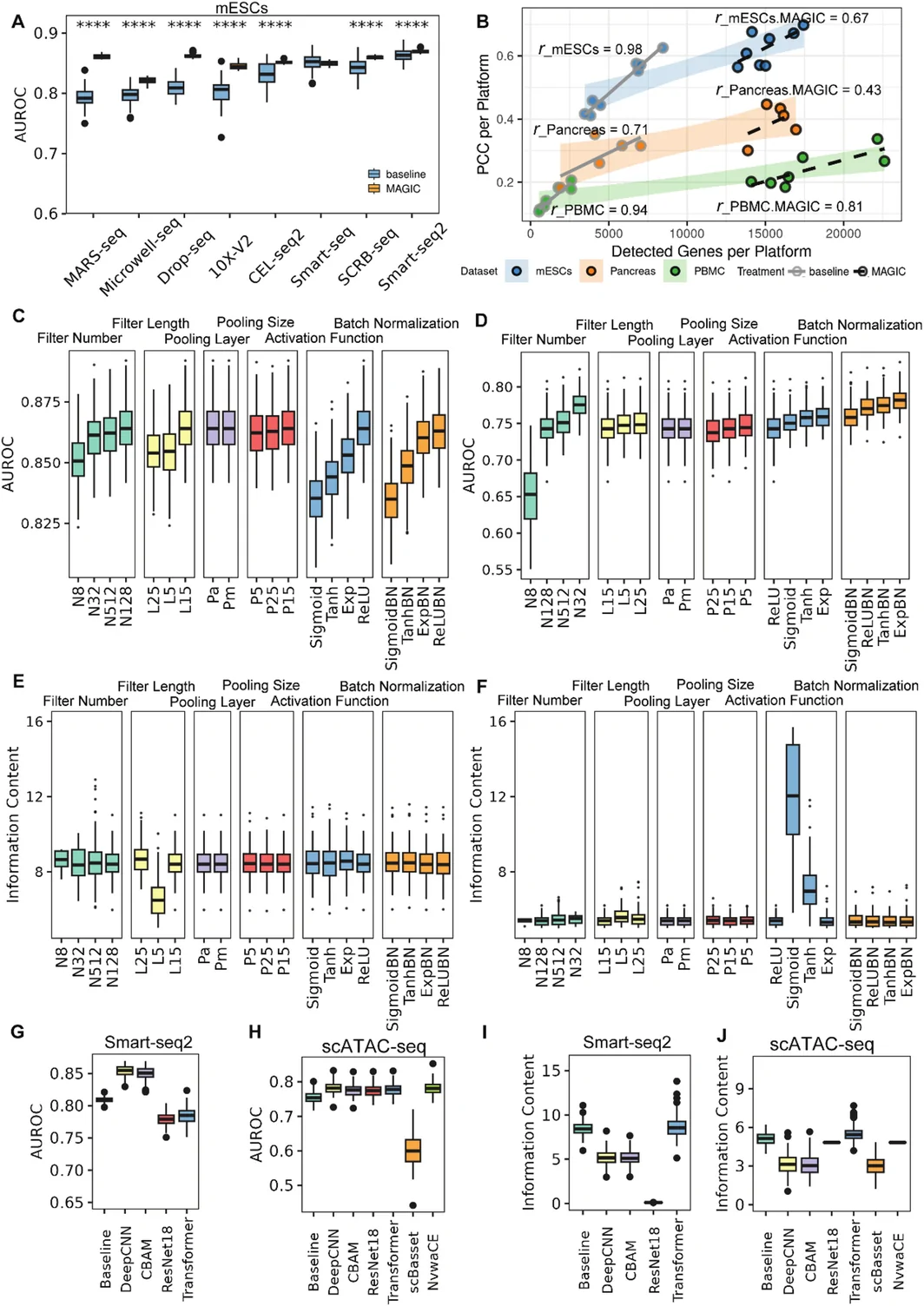
Comparative analysis of sequence‐based deep learning models in single‐cell genomics. (A) AUROC performance of the baseline CNN model using raw and MAGIC‐imputed gene expression across eight sequencing platforms in the mESCs dataset. Statistical significance was determined by two‐sided paired Wilcoxon signed‐rank test; all significant after BH‐FDR correction (****p < 0.0001; only significant comparisons are labelled, *q* < 0.05). (B) Pearson correlation between transcriptomic density (average genes detected per platform) and model regression performance across three biological systems (mESCs, human PBMCs, and human pancreas). Regression lines and 95% confidence intervals (shaded regions) are shown for both raw and MAGIC‐imputed data. (C, D) Hyperparameter benchmarking of predictive accuracy (AUROC) across six architectural dimensions for scRNA‐seq (C) and scATAC‐seq data (D). Parameters include filter number (*N*), filter length (*L*), pooling type (Pa: Average, Pm: Max), pooling size (P5–P25), activation function, and Batch Normalization (BN). (E, F) Boxplots showing the Information Content (IC) scores of the first‐layer filter across identical hyperparameter settings for scRNA‐seq (E) and scATAC‐seq (F). (G, H) Performance comparison (AUROC) of advanced model architectures against the baseline CNN for scRNA‐seq (G) and scATAC‐seq (H). Evaluated models include DeepCNN, CBAM, ResNet18, and Transformer, as well as additionally domain‐specific SOTA models (scBasset and NvwaCE). (I, J) Boxplots showing IC scores for advanced architectures for scRNA‐seq (I) and scATAC‐seq (J). Evaluated models include DeepCNN, CBAM, ResNet18, and Transformer, as well as additionally domain‐specific SOTA models (scBasset and NvwaCE).

Recognizing that high sparsity in single‐cell data limits performance, we employed MAGIC for gene imputation, which yielded consistent and statistically significant improvements in both classification and regression tasks (Wilcoxon rank sum test, *p* < 0.0001) (Figure 1A; Figure S1A–E, and Table S3). The improvement was particularly pronounced in the PBMCs with average AUROC scores increasing from 0.68 to 0.79, and pancreas with average AUROC scores increasing from 0.73 to 0.83 (Figure S1A,B). Performance improvement in mESC (from 0.82 to 0.85) were more modest (Figure 1A). Our results indicated that MAGIC recover gene expression signals previously obscured by technical dropouts. Supporting this, MAGIC dramatically redistributed gene expression rankings, resulting in an approximately 50% positive classification rate under our median‐based binarization, with the most striking improvement in the PBMC dataset (from 5.3% to 49.9%). Furthermore, sensitivity analysis using alternative quantile and mean thresholds confirmed that while absolute performance metrics vary, the relative rankings of sequencing platforms and model architectures remain robust to the specific choice of threshold (Figure S1F and Table S4). In the regression task, visualizations confirmed severe dropout in the PBMC dataset, which MAGIC effectively mitigated (Figure S2). In contrast, mESCs data from Smart‐seq and Smart‐seq2 demonstrated higher sequencing coverage and lower dropout rates, resulting in a more uniform gene expression distribution (Figure S2). Consequently, the imputation effect of MAGIC on Smart‐seq and Smart‐seq2 was marginal, explaining its limited efficacy in enhancing model performance (Figure 1A; Figure S2). Lower Pearson correlation coefficients (*r*) were observed in the MAGIC‐imputed data than in the raw data (Figure 1B; Figure S1G–I). Specifically, the coefficients for MAGIC‐imputed data (mESCs: *r* = 0.67; PBMCs: *r* = 0.81; pancreas: *r* = 0.43) were reduced relative to raw data (mESCs: *r* = 0.98; PBMCs: *r* = 0.94; pancreas: *r* = 0.71). These results indicated a significant association between sequencing depth and model performance, which diminishes as data approaches saturation. As an imputed benchmark condition, MAGIC accelerates this process by compensating for sparsity, thereby reducing the model's dependency on initial sequencing depth. However, we acknowledge potential imputation‐induced bias, such as over‐smoothing or artificial correlations. Thus, while MAGIC enhances model performance in sparse regimes, these results represent a trade‐off between signal recovery and potential biological distortion, requiring cautious interpretation.

We assessed model's reliability across diverse data source and experimental conditions, including sequencing platform (Platform), technical replicate (Replicate A/B), and individual cell (Single cell). The model consistently achieved high AUROC values (primarily 0.8–0.9) across all conditions (Figure S1J), indicating strong insensitivity to experimental conditions. For a given platform, AUROC scores were slightly higher at the platform level than at the single‐cell level. While some variation was observed between technical replicates (e.g., Replicate A and B in CEL‐seq2), the overall performance remained stable. This reliability underscores the model's suitability for downstream biological interpretation.

To complement our focus on data characteristics, we subsequently shifted to optimizing the model by evaluating key hyperparameters on both predictive performance and interpretability. Our results demonstrate that architectural hyperparameters critically governed model performance, with distinct optimal configurations identified for scRNA‐seq datasets (Smart‐seq2 [6] and 10X Genomics [7] and single‐cell epigenetic data from scATAC‐seq [8]). Firstly, model performance was highly sensitive to the number of filters (*N*). For scRNA‐seq data, both classification AUROC and regression PCC improved as *N* increased from 8 to 128, but declined at *N* = 512, suggesting overfitting (Figure 1C; Figure S3A–C). For scATAC‐seq, the AUROC peaked at *N* = 32 and then declined as *N* increased (Figure 1D). Secondly, filter length (*L*) significantly contributed to the scRNA‐seq performance, with both AUROC and PCC increasing from *L* = 5 to *L* = 15 but dropping at *L* = 25 (Figure 1C; and Figure S3A–C). However, this parameter had a negligible impact on the scATAC‐seq models (Figure 1D). Thirdly, hyperparameters related to the pooling layer (types of Pa and Pm, and sizes of P5, P15, P25) showed no significant impact on predictive performance across any dataset. Fourthly, the choice of activation function profoundly affected model performance. For scRNA‐seq, a clear performance gradient was observed (Sigmoid < Tanh < Exp < ReLU) across classification and regression tasks, with ReLU achieving the best results (Figure 1C; Figure S3A–C). This hierarchy was reversed for scATAC‐seq, where Exp performed best and ReLU was the least effective (Figure 1D). Finally, incorporating Batch Normalization (BN), a technique that stabilizes training and accelerates convergence by standardizing layer inputs, led to varying degrees of improvements in predictive performance across all datasets (Figure 1C,D; Figure S3B,C). Notably, hyperparameters influenced the learned PWMs in first‐layer filters less than predictive accuracy. In scRNA‐seq models, the shortest filter length (*L* = 5) yielded the worst Information Content (IC) scores (Figure 1E; Figure S3D), suggesting an excessively small L setting fails to capture the specificity of sequence patterns. For scATAC‐seq, the Sigmoid activation function produced the highest IC scores (Figure 1F), with learned PWM visualization confirming the learning of more distinct and clear sequence features (Figure S3E–H). Collectively, these findings underscore the necessity of modality‐specific hyperparameter selection to maximize both the accuracy and biological relevance of DL models in single‐cell genomics.

Building upon these baseline optimizations, we sought to determine whether advanced DL architectures (Figure S4A,B) translate into tangible gains in single‐cell genomics. On scRNA‐seq datasets, both DeepCNN (AUROC = 0.856 on Smart‐seq2, 0.797 on 10X Genomics) and CBAM [9] (AUROC = 0.852 on Smart‐seq2, 0.792 on 10X Genomics) outperformed the baseline model (AUROC = 0.809) on Smart‐seq2 and achieved comparable performance to the baseline (AUROC = 0.802) on 10X Genomics dataset (Figure 1G; Figure S4C). The discrepancy may be attributable to the higher dropout rate of 10X Genomics data (Figure S2). In contrast, ResNet18 [10] (AUROC = 0.779 on Smart‐seq2, 0.749 on 10X Genomics) and Transformer [11] (AUROC = 0.784 on Smart‐seq2, 0.688 on 10X Genomics) underperformed the baseline (Figure 1G; Figure S4C), despite their theoretical strengths in facilitating deep network training or capturing long‐range dependencies. Notably, the performance patterns shifted on the scATAC‐seq dataset, where ResNet18 (Figure S4A) and Transformer (Figure S4B) exhibited substantial improvements (Figure 1H), contrary to their results on scRNA‐seq. Specifically, in the ultra‐sparse data regime (100 cells), the baseline model (AUROC = 0.755) performed comparably to advanced models including DeepCNN (AUROC = 0.782), Transformer (AUROC = 0.779), CBAM (AUROC = 0.776), and ResNet18 (AUROC = 0.774). When evaluating SOTA sequence‐based architectures tailored for genomic features, NvwaCE [8] achieved the highest performance (AUROC = 0.783), whereas scBasset showed limited sensitivity under these specific, data‐scarce conditions, delivering the least favourable results regardless of whether the input sequence length was 500 bp (AUROC = 0.600) or its default 1344 bp (AUROC = 0.605). Further IC analysis revealed that the specificity of learned PWMs in first‐layer filters decreased as network depth increased (Figure 1I,J; Figure S4D). These findings suggest that for specific single‐cell modalities, architectural complexity does not inherently guarantee better feature representation and may even hinder the learning of distinct biological features at the initial processing stage.

To further explore the interpretability of these complex models, we analysed the spatial attention weights from the CBAM and Transformer components. Our analysis revealed patterns suggestive of the models prioritizing transcription start site (TSS) regions as critical features for predicting gene expression in both Smart‐seq2 (Figure S4E–I) and 10X Genomics datasets (Figure S5). These distributions appear to align with known biologically plausible regulatory elements. Furthermore, different attention heads in the Transformer model exhibited distinct weighting patterns, which may reflect a potential specialization in different aspects of transcriptional regulation (Figures S4F,G and S5A,B). This observation is consistent with the hypothesis that the model learns a distributed representation of regulatory grammar, although it does not provide direct mechanistic proof. By visualizing the query‐to‐key attention, we investigated the internal interactions within specific gene regulatory sequences (Figures S4H and S5C). For each attention head, putative sequence motifs (7‐bp) were identified based on the highest‐scoring peaks; these motifs likely reflect the sequence characteristics that drive model focus (Figures S4I and S5D). We note, however, that while these attention maps provide qualitative insights into model behaviour, they serve as proxies for feature importance rather than definitive evidence of biochemical mechanisms.

Extending our analysis to handle multiple tasks simultaneously, we developed a practical benchmarking framework for multi‐task learning (MTL) [12, 13] as the decoder of the sequence‐based model. Using a hard parameter‐sharing style [14, 15], we partitioned the hidden layer neurons into shared and task‐specific operations. We emphasize that cell‐type and lineage annotations are used exclusively during training to structure the loss function and parameter‐sharing scheme; during inference, the model receives only DNA sequence as input and has no access to cell labels. The model was trained on the MCA dataset [16] to predict expression across 179,344 single cells, utilizing task grouping organized at either the cell‐lineage (MTL_Lineage) or cell‐type (MTL_Celltype) levels.

Primary predictive performance. First, we assessed the primary task: sequence‐based gene expression prediction. Interestingly, we observed a fundamental trade‐off between structural priors and predictive accuracy (Figure 2A). Specifically, the application of task grouping (MTL_Lineage, MTL_Celltype), which imposes a strong structural prior, led to a decline in AUROC values across all cell lineages or cell types compared to the single‐task baseline. This indicates that while MTL regularizes the model, it may introduce a ‘negative transfer’ effect that slightly reduces per‐gene prediction precision. To address this, we implemented adaptive loss‐weighting strategies via the scMTLoss_level parameter at both cell‐type (Loss_Celltype) and cell‐lineage (Loss_Lineage) levels. These strategies effectively mitigated the optimization bias and achieved improved AUROC values over both the baseline and task‐grouping methods (Figure 2A). These findings underscore that adaptive task balancing could enhance primary predictive performance more effectively than imposing fixed structural constraints.

**FIGURE 2:**
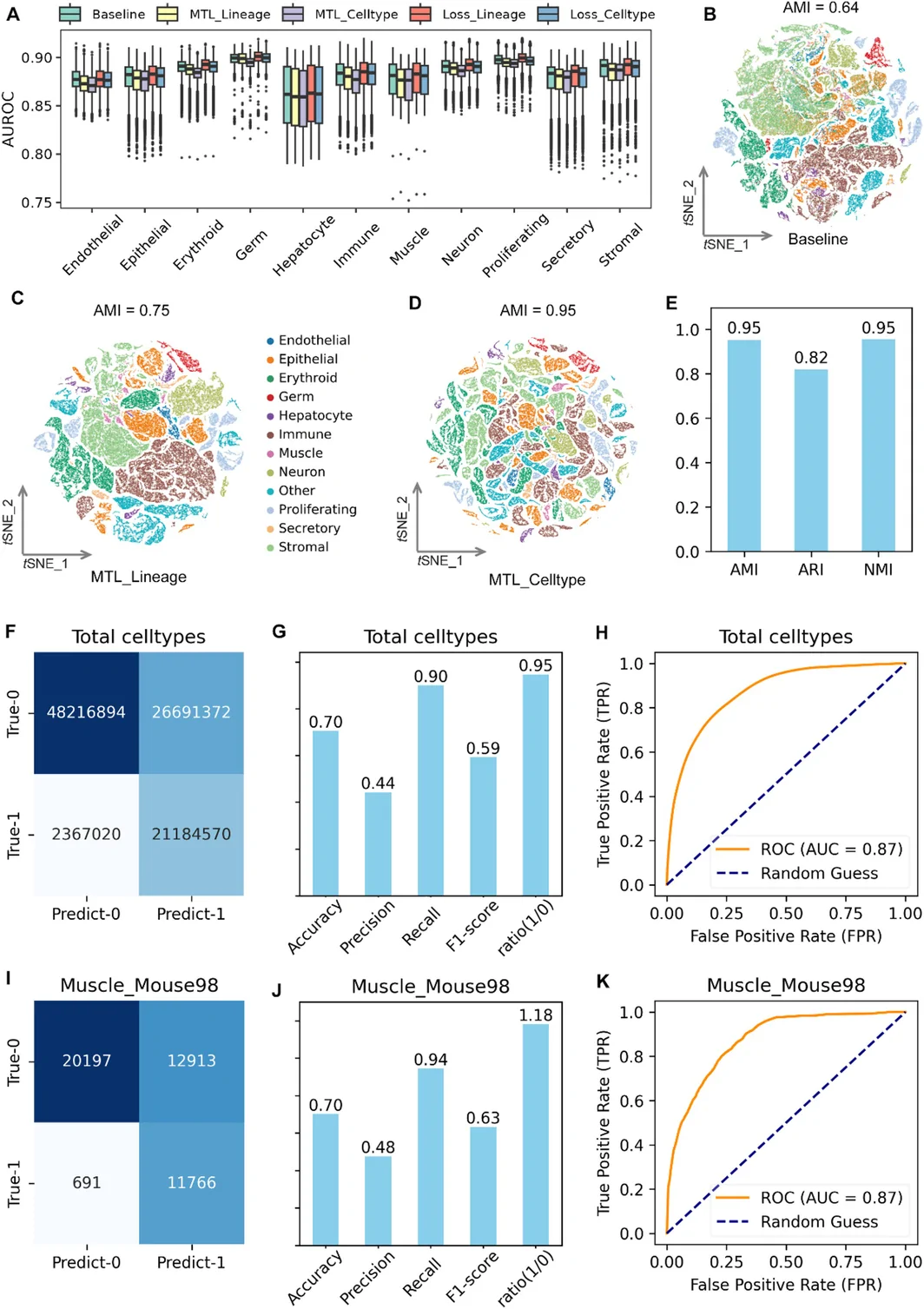
Multi‐Task Learning (MTL) framework enhances cell‐type‐specific prediction and biological representation. (A) Comparison of predictive AUROC values across major cell lineages in the MCA dataset. Boxplots illustrate the performance of the baseline model against four MTL strategies, including hierarchical task grouping (MTL_Lineage and MTL_Celltype) and loss‐weighting variations (Loss_Lineage and Loss_Celltype). (B–D) *t*‐SNE visualizing the predicted cell landscape on the held‐out genes, comparing the baseline model (B), task grouping at the hierarchy of cell‐lineage (MTL_Lineage) (C), and task grouping at the hierarchy of cell‐type (MTL_Celltype) (D). Adjusted Mutual Information (AMI) scores quantify the concordance between unsupervised Louvain clustering (resolution = 0.5) of predicted profiles and ground‐truth biological labels. (E) Quantitative downstream validation of cell identity reconstruction for the MTL_Celltype model, evaluated by AMI, Adjusted Rand Index (ARI), and Normalized Mutual Information (NMI). (F–H) Global evaluation of the sequence‐to‐expression classification task across all cell types. Metrics include the confusion matrix (F), classification metrics (Accuracy, Precision, Recall, F1‐score, and positive/negative ratio) (G), and the Receiver Operating Characteristic (ROC) curve (H). (I–K) Robustness validation on a naturally rare cell type (Muscle_Mouse98, *n* < 100 cells). Evaluation is characterized by the confusion matrix (I), classification metrics (J), and ROC curve (K).

To evaluate whether the MTL framework captures biologically meaningful representations rather than just numerical proximity, we performed cell identity reconstruction as a downstream validation analysis. We used the model's predicted expression profiles to recapitulate the cell atlas and employed adjusted mutual information (AMI), the Adjusted Rand Index (ARI), and Normalized Mutual Information (NMI) to quantify clustering similarity. To ensure robustness and reproducibility, all models were evaluated using a standardized Scanpy pipeline with fixed parameters: *k* = 15 neighbours for graph construction, a Louvain resolution of 0.5, and a t‐SNE perplexity of 30, with a constant random seed (seed = 42). The baseline model achieved moderate clustering concordance (AMI = 0.64, Figure 2B), indicating that sequence‐based predictions alone capture partial cellular heterogeneity. Integrating biological knowledge via MTL significantly improves these downstream results. Specifically, MTL_Lineage increased the AMI score to 0.75 (Figure 2C), while MTL_Celltype achieved the highest scores (AMI = 0.95, ARI = 0.82, and NMI = 0.95) (Figure 2D,E). Notably, the high AMI/ARI/NMI reflects the biological coherence of the predicted expression matrix, not the accuracy of label propagation; the model has no access to cell labels during prediction. For example, while stromal cells, which exhibited substantial internal heterogeneity in the MCA, were prone to clustering into distinct sub‐cell types in both the baseline and MTL_Celltype models (Figure 2D). In contrast, under the MTL_Lineage framework, these cells tended to coalesce into a more unified cluster (Figure 2C). Beyond classification performance, the MTL framework identified 619 candidate rescued genes that were undetected in the original scRNA‐seq data. Functional enrichment analysis revealed that these genes were primarily enriched in pathways related to embryonic stem cell development (Figure S6), suggesting their biological plausibility as potential rescued signals.

Proof‐of‐concept validation on rare cell types. To demonstrate the practical value of integrating the principles derived from our results, such as optimizing data quality and employing MTL strategies, we conducted a proof‐of‐concept validation on rare cell types (Figure S7A,B). We focused on the cell type named Muscle_Mouse98, which was identified as a naturally rare population comprising fewer than 100 cells in the MCA dataset (Figure S7A). We evaluated this primary task using common indicators including confusion matrix, accuracy, precision, recall, F1‐score, and ROC curve analysis. The model demonstrated superior and consistent performance across all metrics (Figure 2F–K). Crucially, we observed perfect clustering consistency (Cluster Coherence Ratio = 1, see Supplementary Methods), indicating that cells predicted by the models reliably aggregated into their original biological cluster (Figure S7C). To further test the model's robustness under controlled benchmarking conditions, we created a more challenging dataset through stratified down‐sampling of the MCA dataset to 10,000 cells, within which we identified 62 artificially rare cell populations (< 100 cells each) (Figure S7B). Applying the same analytical workflow, our model maintained consistent performance (Figure S7D–G), demonstrating its reliability even under conditions of extreme class imbalance. Collectively, our results exhibited remarkable robustness in predicting rare cell populations, underscoring the practical value of MTL in addressing key challenges in single‐cell genomics.

Despite preprocessing differences between datasets, our focus on intra‐dataset platform rankings avoids cross‐dataset confounding. As normalization preserves sparsity, ‘detected gene count’ remains a reliable metric. Our comparative analysis thus establishes a systematic framework for selecting and optimizing DL models in single‐cell genomics, yielding three central principles. First, model selection should be tailored to data modality and quality (e.g., sparsity and detection rates). Rather than a universal hierarchy, architectural performance is context‐dependent. While high‐capacity models (e.g., Transformers, Nvwa) excel in information‐rich scRNA‐seq, fine‐tuned baseline CNNs provide superior robustness in ultra‐sparse scATAC‐seq regimes with severe sample limitations (e.g., 100‐cell limits). Conversely, large‐scale frameworks like scBasset require exceeding critical data thresholds to avoid significant performance degradation. Second, complexity must be balanced against interpretability and efficiency. Increasing network depth or deploying global attention layers yields diminishing returns for standard accessibility prediction while escalating GPU overhead. Crucially, simpler convolutional backbones maintain sharper inductive biases, facilitating clearer feature attribution and sequence pattern identification compared to heavily parameterized counterparts. Third, MTL improves generalization, particularly for rare cell types. Using standard configurations, our results show that architectural complexity doesn't guarantee performance. Although future work will explore exhaustive hyperparameter tuning, lightweight CNNs remain highly competitive and robust baselines for single‐cell sequence prediction. These principles remain critical as large language models (LLMs) emerge with massive‐scale data integration. While models like AlphaGenome [17] (450 M parameters) excel at capturing distal interactions (1 Mb), our benchmark focuses on local regulatory grammar (500 bp–10 kb), where specialized models often deliver more efficient and interpretable solutions for well‐defined tasks with limited data [18, 19, 20]. Directly comparing from‐scratch CNNs against resource‐intensive foundation models would conflate architectural design with massive compute advantages, undermining benchmark fairness. We thus treat LLMs as a complementary paradigm rather than a direct baseline. Ultimately, the future of single‐cell genomics lies in a complementary ecosystem where foundational and specialized models coexist to drive research forward.

## Author Contributions

Guoxia Wen (Methodology, Data Curation, Writing), Jiaqi Li (Methodology, Data Curation, Writing), Hanyu Wu (Methodology, Data Curation), Jialing Fang (Methodology), Yuting Fu (Methodology, Data Curation), Mengmeng Jiang (Data Curation), Yuqing Mei (Methodology), Rui Xu (Data Curation), Yuxuan Du (Data Curation), Yuxuan Du (Data Curation), Siran Chu (Data Curation), Guoji Guo (Conceptualization, Funding acquisition, Supervision, Writing), Xiaoping Han (Supervision), and Jingjing Wang (Conceptualization, Funding acquisition, Supervision, Writing).

## Funding

This research was supported by the National Natural Science Foundation of China (Number 32570784 to J.W., Number 32330061 to G.G.); the Zhejiang Province Vanguard Goose‐Leading Initiative (Number 2025C01114 to G.G.); the Funds for Creative Research Groups of China (Number T2121004 to G.G.).

## Ethics Statement

The authors have nothing to report.

## Consent

The authors have nothing to report.

## Conflicts of Interest

The authors declare no conflicts of interest.

## Supporting information


**Figure S1:** Predictive performance of the CNN model on the different scRNA‐seq technologies.
**Figure S2:** Scatter plot of actual and predicted gene expression values from the logistic regression analysis trained on the mESCs dataset, Human PBMCs datasets and Pancreas dataset.
**Figure S3:** Benchmark of hyper‐parameters on predictive performance of the CNN model.
**Figure S4:** The exploration of advanced model architectures.
**Figure S5:** The exploration of Transformer architectures in the 10X genomics platform.
**Figure S6:** Function enrichment on genes from MCA embryonic dataset that predicted to expressed but not detected by scRNA‐seq.
**Figure S7:** Evaluation of the CNN model with MTL framework on rare cell types.
**Table S1:** Characteristics of different single‐cell sequencing methods used in the study.
**Table S2:** Performance details of scRNA‐seq methods for the CNN model.
**Table S3:** Statistical comparison of baseline and MAGIC‐imputed data across scRNA‐seq platforms.
**Table S4:** Summary statistics of normalized expression values across three datasets.

## Data Availability

The data that support the findings of this study are openly available in figshare at https://doi.org/10.6084/m9.figshare.33069326. The code used in this work can be downloaded from https://github.com/ggjlab/benchmark_DL_CNN. All the used model architectures were provided in BaselineModel.py. We also provided the preprocessing scripts, model configurations, training and evaluation pipelines, figure‐generation scripts, and environment files.
