# Supplementary material for: A Comprehensive Comparative Analysis of Sequence‐Based Deep Learning Models for Single‐Cell Genomics
Source: Cell Prolif. 2026 Aug 23;59(10):e70274. doi: 10.1111/cpr.70274 (PMC13500174; doi:10.1111/cpr.70274)
Supplement: Supplementary file 1 [file CPR-59-e70274-s001.docx]

**FIGURE S1. Predictive performance of the CNN model on the different scRNA-seq technologies.**

**(**A, B**)** AUROC performance of the baseline CNN model using raw and MAGIC-imputed gene expression across different sequencing platforms in the Human PBMCs datasets (A) and Pancreas dataset (B).

(C-E) Pearson correlation coefficient (PCC) of the baseline CNN model using raw and MAGIC-imputed gene expression across different sequencing platforms in the mESCs dataset (C), Human PBMCs datasets (D) and Pancreas dataset (E).

(F) Sensitivity of platform performance rankings to binarization thresholds in mESCs. Boxplots show per-cell AUROC distributions for baseline (threshold = 0.001) and MAGIC-imputed data under four alternative thresholds: the same threshold to baseline (0.001), median (0.036), mean (1.305), and 75th percentile (1.663).

(G-I) PCC of the predictive performance of the CNN model and the average detected number of genes of different sequencing platforms in baseline and MAGIC group in the mESCs dataset (G), Human PBMCs datasets (H) and Pancreas dataset (I).

(J) Robustness validation of the CNN model across diverse data source and experimental conditions, including sequencing platform (Platform), technical replicate (Replicate A/B), and individual cell (Single cell). Statistical significance was assessed using the two-sided Wilcoxon signed-rank test, paired by cell. P-values were adjusted using the Benjamini-Hochberg false discovery rate (FDR) method within each dataset. All reported comparisons remain significant at q < 0.05 after FDR correction. * p < 0.05, ** p < 0.01, *** p < 0.001, **** p < 0.0001.

**FIGURE S2.** Scatter plot of actual and predicted gene expression values from the logistic regression analysis trained on the mESCs dataset, Human PBMCs datasets and Pancreas dataset.

**FIGURE S3.** **Benchmark of hyper-parameters on predictive performance of the CNN model**.

(A) AUROC performance of the baseline CNN model across various hyper-parameters trained on the 10X datasets.

(B-C) PCC performance of the baseline CNN model with different hyper-parameters trained on the Smart-seq2 dataset (B) and 10X datasets (C).

(D) Information content scores of the learned PWMs derived from first-layer filters across different hyper-parameters settings trained on the 10X datasets.

(E-H) Comparison of PWMs learned by the first-layer filters trained on scATAC-seq with the different activation functions: ReLU (E), Sigmoid (F), Tanh (G) and Exp (H).

**FIGURE S4. The exploration of advanced model architectures.**

(A, B) Illustration of Residual blocks (A) and Transformer Encoder (B).

(C) Performance of models to predict single-cell expression by AUROC trained on 10X-V2 dataset.

(D) Information content of models trained on 10X-V2 dataset.

(E) The average position attention of CBAN model trained on Smart-seq2 dataset.

(F) The average position attention of Transformer model trained on Smart-seq2 platform. x-axis indicated the relative sequence position from gene TSS location before attention layer.

(G) The different position attention heads of Transformer model trained on Smart-seq2 platform. x-axis indicated the relative sequence position from gene TSS location before attention layer.

(H) The self-attention showed the top interaction from sequence to sequence.

(I) The enrichment of motifs of sequences with the highest attention interaction of distinct attention heads.

**FIGURE S5. The exploration of Transformer architectures in the 10X genomics platform.**

(A) The average position attention of Transformer model trained on 10X Genomics dataset. x-axis indicated the relative sequence position from gene TSS location before attention layer.

(B) The different position attention heads of Transformer model trained on 10X dataset. x-axis indicated the relative sequence position from gene TSS location before attention layer.

(C) The self-attention showed the top interaction from sequence to sequence.

(D) The enrichment of motifs of sequences with the highest attention interaction of distinct attention heads.

**FIGURE S6.** Function enrichment on genes from MCA embryonic dataset that predicted to expressed but not detected by scRNA-seq.

**FIGURE S7.** **Evaluation of the CNN model with MTL framework on rare cell types.**

(A) Distribution of cell numbers among 98 cell types in the original MCA data.

(B) Distribution of cell numbers among 98 cell types after stratified down-sampling on the MCA dataset to a total of 10,000 cells.

(C) Cluster Coherence Ratio of genes between rare cell type (musle_mouse98) and its predicted cluster.

(D) Cluster Coherence Ratio between various rare cell types in the down-sampled dataset and the corresponding predicted cell clusters.

(E) Confusion matrix of model performance on rare cell types within the down-sampled dataset.

(F) Statistic metrics evaluating model performance in rare cell types.

(G) ROC curve of model for rare cell types.

**Table S1.** Characteristics of different single-cell sequencing methods used in the study

|  | Principle | Features | Limitations | Ref. |
| --- | --- | --- | --- | --- |
| CEL-seq2 | Based on linear amplification of in vitro transcription using barcoded UMI labeling of individual cells, detected by second-generation sequencing. | High sensitivity;  Multiple sample mixing; | 3' end preference;  Low throughput; | (Hashimshony et al., 2016) |
| Drop-seq | Microfluidic droplet technology, where cells are encapsulated with magnetic beads with barcodes in oil droplets for single-cell isolation and mRNA labeling. | Ultra-high throughput;  UMI labeling; | Low capture efficiency;  High doublet rate; | (Macosko et al., 2015) |
| MARS-seq | Combinatorial indexing technique (cell & molecular barcoding) by FACS sorting and in vitro transcription amplification. | High accuracy;  Suitable for rare cells; | Low throughput;  Complex experimental process; | (Jaitin et al., 2014; Soumillon et al., 2014) |
| SCRB-seq | Amplification technology based on cell barcoding and Template Switching. | High sensitivity;  Low cost; | Medium throughput;  3' end preference; | (Soumillon et al., 2014) |
| Smart-seq / Smart-seq2 | Full-length transcriptome amplification (based on template conversion and PCR) to detect full-length mRNA sequences. | Full-length coverage;  Full-length coverage; | Low throughput; | (Picelli et al., 2014; Ramsköld et al., 2012; Wang et al.) |
| Microwell-seq | Microtiter plate-based barcode labeling technology combined with high-throughput sequencing. | Ultra-high throughput;  Flexible labeling; | Low capture efficiency;  Platform-dependent; | (Han et al., 2018) |
| 10X Genomics | Microfluidics & molecular barcoding. | High- throughput; | 3' end preference; High dropout; | (Wang et al.) |

**Table S2.** Performance details of scRNA-seq methods for the CNN model

| Datasets | Platforms | No. of  cells | No. of  detected genes | PCC | AUROC |
| --- | --- | --- | --- | --- | --- |
| mESCs | 10X-V2 | 100 | 4467 | 0.44 | 0.80 |
|  | MAGIC-10X-V2 | 100 | 13,667 | 0.57 | 0.85 |
|  | CEL-seq2 | 71 | 7391 | 0.58 | 0.83 |
|  | MAGIC-CEL-seq2 | 71 | 11,145 | 0.66 | 0.85 |
|  | Drop-seq | 76 | 3745 | 0.45 | 0.81 |
|  | MAGIC-Drop-seq | 76 | 8876 | 0.62 | 0.86 |
|  | MARS-seq | 65 | 3697 | 0.43 | 0.79 |
|  | MAGIC-MARS-seq | 65 | 8766 | 0.59 | 0.86 |
|  | Microwell-seq | 100 | 3445 | 0.47 | 0.80 |
|  | MAGIC-Microwell-seq | 100 | 8746 | 0.60 | 0.82 |
|  | Smart-seq | 130 | 7402 | 0.61 | 0.85 |
|  | MAGIC-Smart-seq | 130 | 10,965 | 0.69 | 0.85 |
|  | Smart-seq2 | 157 | 8741 | 0.63 | 0.86 |
|  | MAGIC-Smart-seq2 | 157 | 11,764 | 0.70 | 0.87 |
|  | SCRB-seq | 84 | 7351 | 0.59 | 0.84 |
|  | MAGIC-SCRB-seq | 84 | 11,153 | 0.69 | 0.86 |
|  | Average_baseline | 95 | 5780 | 0.53 | 0.82 |
|  | Average_MAGIC | 95 | 10635 | 0.64 | 0.85 |
|  | Average_total | 95 | 8208 | 0.58 | 0.84 |
| Human  PBMCs | 10X-V2 | 583 | 1004 | 0.14 | 0.70 |
|  | MAGIC-10X-V2 | 583 | 12,494 | 0.22 | 0.80 |
|  | 10X-V3 | 583 | 1772 | 0.18 | 0.69 |
|  | MAGIC-10X-V3 | 583 | 13,513 | 0.28 | 0.82 |
|  | CEL-Seq2 | 583 | 2604 | 0.21 | 0.68 |
|  | MAGIC-CEL-Seq2 | 583 | 17,101 | 0.34 | 0.79 |
|  | Drop-seq | 583 | 866 | 0.12 | 0.66 |
|  | MAGIC-Drop-seq | 583 | 11,722 | 0.18 | 0.79 |
|  | inDrops | 583 | 545 | 0.11 | 0.68 |
|  | MAGIC-inDrops | 583 | 10,099 | 0.20 | 0.77 |
|  | Seq-Well | 583 | 591 | 0.11 | 0.70 |
|  | MAGIC-SeqWell | 583 | 10,588 | 0.20 | 0.79 |
|  | Smart-seq2 | 583 | 2606 | 0.18 | 0.66 |
|  | MAGIC-Smart-seq2 | 583 | 16931 | 0.27 | 0.78 |
|  | Average_baseline | 583 | 1427 | 0.15 | 0.68 |
|  | Average_MAGIC | 583 | 13,207 | 0.24 | 0.79 |
|  | Average_total | 583 | 7317 | 0.20 | 0.74 |
| Human  Pancreas | CEL-Seq2 | 583 | 4109 | 0.35 | 0.75 |
|  | MAGIC-CEL-Seq2 | 583 | 10,441 | 0.45 | 0.82 |
|  | fluidigmc1 | 583 | 7040 | 0.32 | 0.75 |
|  | MAGIC-fluidigmc1 | 583 | 8457 | 0.43 | 0.83 |
|  | inDrops | 583 | 1916 | 0.18 | 0.69 |
|  | MAGIC-inDrops | 583 | 8457 | 0.30 | 0.80 |
|  | SMARTer | 583 | 4402 | 0.26 | 0.73 |
|  | MAGIC-SMARTer | 583 | 10,021 | 0.37 | 0.84 |
|  | Smart-seq2 | 583 | 5848 | 0.32 | 0.75 |
|  | MAGIC-Smart-seq2 | 583 | 11,112 | 0.41 | 0.84 |
|  | Average_baseline | 583 | 4663 | 0.29 | 0.73 |
|  | Average_MAGIC | 583 | 9698 | 0.39 | 0.83 |
|  | Average_total | 583 | 7180 | 0.34 | 0.78 |

**Table S3. Statistical comparison of baseline and MAGIC-imputed data across scRNA-seq platforms.**

| Dataset | Platform | n_  baseline | n_  magic | p_  AUROC | q_  AUROC | p_  PCC | q_  PCC |
| --- | --- | --- | --- | --- | --- | --- | --- |
| mESCs | 10X-V2 | 100 | 100 | 4.08e-33 | 3.26e-32 | 7.36e-33 | 1.47e-32 |
|  | CEL-seq2 | 71 | 71 | 3.22e-09 | 3.68e-09 | 8.52e-25 | 9.74e-25 |
|  | Drop-seq | 76 | 76 | 1.94e-26 | 5.17e-26 | 1.94e-26 | 2.59e-26 |
|  | MARS-seq | 65 | 65 | 8.03e-23 | 1.61e-22 | 1.11e-22 | 1.11e-22 |
|  | Microwell-seq | 100 | 100 | 3.21e-29 | 1.28e-28 | 2.56e-34 | 6.83e-34 |
|  | Smart-seq | 130 | 130 | 0.0539 | 0.0539 | 3.79e-44 | 1.52e-43 |
|  | Smart-seq2 | 157 | 157 | 2.86e-12 | 3.81e-12 | 1.03e-51 | 8.24e-51 |
|  | SCRB-seq | 84 | 84 | 8.75e-16 | 1.40e-15 | 4.56e-29 | 7.30e-29 |
| Human  PBMCs | 10X-V2 | 583 | 583 | 2.91e-187 | 1.02e-186 | 3.99e-160 | 5.59e-160 |
|  | 10X-V3 | 583 | 583 | 5.00e-192 | 3.50e-191 | 7.33e-180 | 5.13e-179 |
|  | CEL-Seq2 | 583 | 583 | 1.90e-183 | 3.32e-183 | 2.55e-174 | 8.92e-174 |
|  | Drop-seq | 583 | 583 | 4.63e-186 | 1.08e-185 | 5.95e-112 | 5.95e-112 |
|  | inDrops | 583 | 583 | 2.33e-132 | 2.33e-132 | 8.03e-169 | 1.87e-168 |
|  | Seq-Well | 583 | 583 | 9.38e-152 | 1.09e-151 | 2.84e-136 | 3.31e-136 |
|  | Smart-seq2 | 583 | 583 | 1.59e-171 | 2.23e-171 | 2.82e-162 | 4.93e-162 |
| Human  Pancreas | CEL-Seq2 | 583 | 583 | 1.09e-189 | 1.09e-189 | 9.75e-140 | 1.22e-139 |
|  | fluidigmc1 | 583 | 583 | 5.66e-192 | 1.41e-191 | 4.84e-189 | 2.42e-188 |
|  | inDrops | 583 | 583 | 2.33e-190 | 2.91e-190 | 1.29e-176 | 3.22e-176 |
|  | SMARTer | 583 | 583 | 5.00e-192 | 1.41e-191 | 5.26e-108 | 5.26e-108 |
|  | Smart-seq2 | 583 | 583 | 2.18e-190 | 2.91e-190 | 1.81e-172 | 3.02e-172 |

*Statistical significance was assessed using the two-sided Wilcoxon signed-rank test, paired by cell. P-values were adjusted using the Benjamini-Hochberg false discovery rate (FDR) method within each dataset. All reported comparisons remain significant at q < 0.05 after FDR correction.*

**Table S4 Summary statistics of normalized expression values across three datasets**

|  | mESCs | mESCs MAGIC | PBMC | PBMC MAGIC | Pancreas | Pancreas MAGIC |
| --- | --- | --- | --- | --- | --- | --- |
| Median value | 0.000 | 0.036 | 0.000 | 0.027 | 0.000 | 0.130 |
| Mean value | 1.269 | 1.305 | 0.425 | 0.395 | 0.515 | 0.523 |
| 25^th^ value | 0.000 | 0.000 | 0.000 | 0.000 | 0.000 | 0.001 |
| 75^th^ value | 0.000 | 1.663 | 0.000 | 0.300 | 0.000 | 0.687 |

## **Materials and Methods**

### **1 Single-cell data collection**

To analyze the effect of different scRNA-seq methods on the performance of DL models, we curated and integrated one mouse and two human publicly available scRNA-seq datasets.

1) Mouse Embryonic Stem Cells (mESCs): We first obtained differential gene expression (DGE) matrices from NCBI GEO under accession number GSE75790, which comprise data from 583 mESCs generated using six prominent scRNA-seq methods, including CEL-seq2 (71 cells) (Hashimshony et al., 2016), Drop-seq (76 cells) (Macosko et al., 2015), MARS-seq (65 cells) (Jaitin et al., 2014), SCRB-seq (84 cells) (Soumillon et al., 2014), Smart-seq (130 cells) (Ramsköld et al., 2012) and Smart-seq2 (157 cells) (Picelli et al., 2014). And each technology has two experimental replicates (Ziegenhain et al., 2017). Additionally, we randomly selected 100 cells from the mouse embryonic stem cell dataset, named EmbryonicStemCell.CJ7 (accession number GSM2906414) sourced from Mouse Cell Atlas (MCA) by Microwell-seq (accession number GSE108097), and 100 cells from mESCs dataset sequenced by 10X Genomics (accession number GSM4017119). These datasets from relatively homogeneous mESCs were merged based on common genes, resulting in a final dataset comprising 783 cells and 22,433 genes. The variable cell numbers per platform (65–157 cells) in mESCs can reflect realistic platform throughputs and validate the independence of baseline model’s performance with cell number.

2) Human Peripheral Blood Mononuclear Cells (PBMCs): This dataset was sequenced by seven different scRNA-seq protocols: 10X Chromium (v2), 10X Chromium (v3), CEL-Seq2, Drop-seq, inDrops, Seq-Well, and Smart-seq2. The dataset is available at <https://singlecell.broadinstitute.org/single_cell/study/SCP424/>single-cell-comparison-pbmc-data. We randomly selected 583 cells from each technology, resulting in a final dataset comprising 4,081 cells and 26,374 common genes.

3) Human Pancreas: This dataset was downloaded from <https://figshare.com/ndownloader/files/24539828>. It was sequenced by six scRNA-seq methods: CEL-Seq2, inDrops, fluidigmc1, SMARTer, and Smart-seq2. Similarly, we randomly selected 583 cells from each method and generated a final dataset with 3,498 cells and 19,093 common genes.

We also collected a public mouse scATAC-seq data, which was generated using UUATAC-seq protocol (Han et al., 2025), For the purpose of this study, which was to provide a controlled and computationally efficient benchmark for hyperparameter tuning, we filtered the data to exclude brain tissue and performed random downsampling to obtain 100 cells for training and validation.

To compared the cellular specificity of task grouping structure at different hierarchical levels, we downloaded datasets from MCA dataset (accession number GSE GSE108097), which included 179,344 single cells sequencing data by using Microwell-seq and provided three levels of hierarchical annotation based on cell ontology, including single-cell, cell-type, and cell-lineage.

### **2 Single-cell data preprocessing**

For the mESCs and human PBMCs datasets, the total counts per cell were normalized to 100,000, followed by log-transformation. The function of ‘normalize_total’ and ‘log1p’ of Scanpy (v1.8.1) (Wolf et al., 2018) was applied for the processing. For Human Pancreas datasets, where raw counts were unavailable, the pre-normalized data was directly used from the provided h5ad file. Specifically, the Pancreas dataset (a heterogeneous tissue) was processed with scran + log1p by the original authors. Although the normalization method (scran+ log1p) is functionally analogous to the normalize_total+ log1p transformation we applied to the mESC and PBMC count matrices, we agree that the differences in preprocessing could introduce variations in expression scale and MAGIC input conditions. Finally, matrices from different technologies were integrated by retaining only the intersecting genes for the downstream analysis.

In order to analyze the impact of single-cell sparsity on model prediction, we performed gene imputation on the normalized expression matrices using the MAGIC algorithm (v2.0.3) (van Dijk et al., 2018) with its default parameters. This step aimed to recover gene expression dynamics that might be obscured by technical dropout events.

The preparation of the expression matrix was tailored to the specific DL task. For the regression task, genes were deemed expressed if their normalized expression values exceed 0. The continuous expression matrix was input directly into the model. For the classification task, the continuous expression matrix was binarized. Gene expression values were assigned to “expressed” (labeled 1) or “non-expressed” (labeled 0) using the median expression value as the threshold. The dataset-specific median thresholds were as follows: in the mESCs dataset, the cutoff value was 0.001 for the baseline data, and 0.036 for the MAGIC processing; in the PBMCs dataset, the cutoff value was 0.001 for the baseline data, and 0.027 for the MAGIC processing; in the Human Pancreas datasets, the cutoff value was 0.001 for the baseline data, and 0.130 for the MAGIC processing.

### **3 Baseline model architecture**

Our CNN architecture transforms raw input (DNA sequence one-hot encoding with 4 channels) into high-dimensional feature representations to predict each gene’s expression or genomic window’s accessibility at the single-cell levels and consists of the following key components.

Convolution 1d Layer: Consisting of 128 filters with size of 15, Rectified Linear Unit (ReLU) activation function, and average-pooling layer with size of 15.

Convolution 2d Layer: Consisting of 256 filters with size of 3, batch normalization (BN), ReLU, and average-pooling layer with size of 3, followed by flatten layer.

Decoder Layer: Consisting of linear transformations, a BN layer, ReLU and a dropout layer with a dropout rate of 0.5 to reduce overfitting.

Predictor Layer: Integrating former feature into the a fully connected feed-forward network (FFN) block to produce prediction for the cell expression state and scaled the prediction to expression probability by a Sigmoid activation function, enabling muti-task learning and multi-label classification.

The choice of a two-layer CNN as our baseline is directly informed by our previously established model, Nvwa, which demonstrate that a hierarchical two-layer structure is the minimal effective architecture for sequence-to-function tasks in genomics. Layer 1 (Motif Detection): the first layer (128 filters, kernel size 15) captures local regulatory elements equivalent to Position Weight Matrices (PWMs). Layer 2 (Regulatory Syntax): The second layer (256 filters, kernel size 3) serves to integrate these motif signals, capturing the spatial dependencies and regulatory syntax between elements. By utilizing this standard two-layer configuration, we ensure our baseline is both biologically representative and computationally comparable to state-of-the-art frameworks.

### **4 Model input**

The regulatory sequences of genes were considered as 10 kb sequence centered at the transcription start site (TSS). These promoter sequences were obtained according to gene coordinates using pysam, a Python interface to samtools. Chromatin accessibility peaks from scATAC-seq were normalized to 500 bp intervals. Expect scBasset, all other models were evaluated using the same input length (500 bp for standard peak-based architectures) with identical genome-alignment and padding protocols to maintain consistency across comparative metrics. Specially, scBasset was originally designed for a native input window of 1,344 bp to capture sufficient sequence context. To test its adaptability and ensure a fair comparison under uniform peak resolutions, we also evaluated it using resized 500 bp inputs. Both evaluations revealed performance constraints under the specific sparsity and architectural dynamics of our scATAC-seq benchmark.

All input DNA sequences were encoded using the one-hot encoding scheme. In this study, genome sequences were based on the mouse GRCm38 assembly and the and human GRCh38 assembly.

To strictly prevent overlap between the training and testing sets, we held out all genes and peaks located on chromosome 8 (chr8) as the test set. Genes and peaks on the other chromosomes were randomly split into training and validation sets at a 9:1 ratio. Finally, the corresponding label (a gene expression vector or an accessible peak vector) and was paired with its input one-hot encoding regulatory sequence to generate the complete dataset for DL model.

### **5 Model training**

A minibatch of N (N=128) was fed through the network, generating predictions for each individual cell state. To train the model, we minimized the objective function by standard backpropagation, which optimized the learnable parameters using Adam optimizer. To avoid overfitting, we stopped training early when the model converged on the validation set. All models were implemented using PyTorch 1.5.0+cu101, CUDA 10.1, the Anaconda3 distribution of Python and trained on computer cluster equipped four NVIDIA A100 Tensor Core GPUs.

To ensure reproducibility, a fixed random seed of 12 was applied to all stochastic operations including weight initialization and data shuffling via the NvTK framework (NvTK.set_random_seed() and NvTK.set_torch_seed()). Additionally, torch.backends.cudnn.deterministic was set to ‘True’ to enforce deterministic CUDA operations, and all DataLoaders used num_workers=0 to eliminate multi-process randomness. Under these fully deterministic settings, a single representative training run was performed for each experimental condition, as repeated runs with identical seeds and deterministic backends yield mathematically equivalent results. Data shuffling during training is deterministic due to fixed random seed initialization. Validation and test data were processed without shuffling to ensure consistent evaluation. All reported metrics (PCC, SCC, AUROC, AUPR) represent results from a single deterministic training run, with boxplots illustrating the distribution across 3,000 cell types or tasks.

### **6 Model interpretation**

To empirically evaluate whether CNN model learns biologically meaningful features, we analyzed the filters from its first-layer convolution. Each filter was extracted and converted into a position weight matrix (PWM) to represent the sequence pattern it detects. This was done by aggregating nucleotide sequences from the input data that strongly activated the filter, using an activation threshold of 0.9 (i.e., only nucleotides that activated the filter to at least 90% of its maximum value were counted). These filters typically correspond to important sequence features (such as TF binding sites, promoter signals, etc.).

We then quantified the specificity of each filter by calculating the Information Content (IC) of its corresponding PWM. IC measures the deviation of the nucleotide distribution at each position from a uniform background model, effectively representing the "information gain" or specificity of the motif.

$$IC(M,B)=\sum_{i=1}^{m} \sum_{j=1}^{4} M_{i,j}\frac{M_{i,j}}{B_{j}}$$

where M and B indicate the PWM (4 × m PWM, m = 7 in our model) and background, respectively; M_i,j_ is the probability of base j at position i in M; and B_j_ is the probability of the same letter in the background model.

A high IC value indicates that a convolutional filter has learned a consistent, information-rich sequence pattern. While such patterns are necessary for representing true biological motifs like TFBS, a high IC alone is not sufficient evidence for a direct biological match; additional validation (e.g., motif enrichment analysis against known databases) is required for definitive biological annotation. Consequently, high-IC filters were prioritized as those capturing the most specific and potentially functional signatures within the genomic data.

### **7 Model performance evaluation**

Model performance was evaluated separately for classification and regression tasks, as specified by the –tasktype parameter including “binary_classification” and “regression”.

For binary classification, the model’s output gene expression probability matrix was binarized based on the median value of the predictions. The classification performance was assessed using sklearn.metrics module by calculating the confusion matrix, Accuracy, Precision, F1 score, and the Area Under the Receiver Operating Characteristic curve (AUROC).

For regression analysis, the model’s performance was quantified by calculating the Pearson correlation coefficient (PCC) between the input expression matrix and the predicted expression matrix.

### **8 Performance benchmarking of varying experimental conditions for the CNN model**

To evaluate the robustness of the model under varying experimental conditions, we performed predictions on the mESCs dataset under three distinct grouping strategies using the --subset_task_byparameter. First, we modeled all cells collectively within each sequencing platform (Platform) to assess cross-platform consistency. Second, we modeled cells separately for each experimental replicate (Replicate A/B) within a platform to gauge technical reproducibility. Third, we generated a prediction for each individual cell (Single cell) to examine cell-level variation. The resulting AUROC values across all platforms and replicates were used to validate the robustness of the models.

### **9 Performance benchmarking of hyper-parameters for the CNN model**

The choice of hyperparameters has an important impact on the performance of DL models. By adjusting the hyperparameters, the structure of the model and the training process can be optimized to improve the prediction and generalization ability of the model. Hence, we benchmarked different hyper-parameters of the CNN model on Smart-seq2, 10X Genomic from mESCs and scATAC-seq datasets from mouse brain. We initialized the baseline CNN model and compared the hyper-parameters of the first layer convolution as follows: (1) the convolutional filter numbers (N), which represents the number of features generated by the convolution, can vary among 8, 32, 128 (Default) and 512; (2) the convolutional filter length (L), which represents the size of the convolutional kernel, can vary between 5 and 15 (Default), 25; (3) the type of pool layer , can vary between “average pooling (Pa)” (Default) and “max pooling (Pm)”, of which Pa calculates the average value of pixels within each pooling region as the output, while Pm selects the maximum value;(4) the pooling size (P), which represents the size of the pooling window and the stride of pooling is equal to the pooling size by default, can vary among 5, 15 (Default) and 25; (5) the activation function, can vary among “ReLU” (Default), “Sigmoid”, “Tanh” and “Exponential” activation; (6) The application of Batch Normalization (BN), which normalizes the mean and standard deviation per dimension across mini-batches, is denoted by 'BN'.

For each hyper-parameter configuration, a single deterministic training run was performed with the fixed random seed and deterministic settings described in the Model training section.

We compared the predictive performance with the Area Under the Receiver Operating Characteristic curve (AUROC) and Pearson Correlation Coefficient (PCC) for classification and regression analysis respectively.

### **10 Performance benchmarking of advanced model architectures**

In addition to the representation learning facilitated by the first-layer convolution, the architectural design of the encoder plays a crucial role in capturing higher-order syntactic rules within the feature map. Several cutting-edge deep learning architectures, such as those incorporating residual connections and attention mechanisms, have demonstrated substantial advancements in the fields of computer vision and natural language processing. However, their application in genomics remains limited. Leveraging above scRNA-seq (10X and Smart-seq2 platforms) and scATAC-seq (UU-ATAC-seq) datasets, we investigated the suitability of various deep learning models for single-cell expression prediction., including deep CNNs, CANs, ResNet, Transformer, scBasset and NvwaCE. Taking the baseline model as starting point, we enhanced the depth of DeepCNN by adding two extra convolutional layers with the filter length of 15. In regards to ResNet, we incorporated the Residual mechanism to assist in building substantially deeper model, while combatting the vanishing gradient problem commonly associate with building deep models. The network depth in the ResNet was 18. Next, we turned to implement the CAN by employing the CBAM. The number of gate channels was 256. Finally, the Transformer encoder was implemented with the Multi-Head Attention mechanism, which excludes the necessity for recurrence and convolutions, instead favoring a more direct approach. The transformer encoder had 2 layers and each layer had 2 heads. The dimension of the feedforward network model was 32. Each architecture variant was trained under identical deterministic conditions with a fixed random seed of 12. For statistical comparison in Figure 1G-H, five independent training runs were performed per architecture using seeds 12, 42, 123, 456, and 789, with results reported as mean ± standard deviation.

In this study, to control variables and focus on the core differences in architecture, the baseline CNN underwent systematic hyperparameter optimization for this task, while the compared advanced models adopted standard or recommended configurations within their respective fields. This design aims to evaluate the potential of these complex architectures in an 'out-of-the-box' scenario, rather than their absolute performance ceiling after equivalent deep tuning. It should be emphasized that the comparison of advanced models in this study is based on their standard configurations. Future work includes conducting a comprehensive hyperparameter search for each architecture to map out its complete performance profile on this task. However, our preliminary results indicate that the complexity of an architecture itself is not a guarantee of performance. In the task of single-cell sequence prediction, a well-understood and lightweight CNN architecture serves as a highly competitive strong baseline.

### **11 MTL framework for predicting cell-type specificity**

In single-cell genomics, downstream analyses prioritize the characterization of cellular heterogeneity. To preserve the hierarchical structure of this heterogeneity for subsequent modeling and interpretation, it is critical to integrate such structural information into predictive frameworks. The MTL framework effectively mitigates noise inherent in single-cell tasks by dynamically leveraging sequence features from both similar and dissimilar cells. Within the MTL architecture, the hidden neurons of the Feedforward Neural Network (FNN) block are structured according to the hierarchical cell annotations. For instance, the initial FNN layer may be partitioned based on cell-type annotations, while subsequent output neurons can be further specialized to the single-cell or task level. Consequently, the FNN’s hidden neurons are systematically organized in alignment with the cell annotation hierarchy.

We used the mean of binary cross entropy loss (BCELoss) as the objective function to measure the discrepancy between each prediction and its corresponding target. In the baseline model, the final objective directly averaged loss of each cell, which can be denoted as: $MTLoss = \frac{1}{N}\sum_{c}^{N} \mathrm{Loss}_{c}$, where c denote each cell/task and N denote the number of cells/tasks. By introducing the prior cell annotation information, the final objective could be balanced according to the hierarchical annotation, which could be denoted as: $MTLoss=\frac{1}{H}\sum_{h}^{H} (\frac{1}{\mathrm{Ch}}\sum_{c}^{\mathrm{Ch}} \mathrm{Loss}_{c})$ and $\sum_{h}^{H} \mathrm{Ch}=N$, where c denotes each cell/task, h denotes each hierarchical annotation, H denote the total number of hierarchical annotation, Ch denote the number of cells/tasks in h and N denote the total number of all cells/tasks. Note the sum of cells in each specific hierarchical annotation should be equal to the total number of all cells.

We applied this framework to the MCA datasets, which provides three hierarchical levels of annotation based on cell ontology. 1) Single-cell level: This corresponds to baseline predictive task and represents the smallest biological and modeling unit, where the model predicts gene expression for individual cells. 2) Cell-type level: Derived from unsupervised clustering, cell types are biologically validated through canonical marker gene expression profiles, allowing the model to learn cell-type-specific regulatory patterns. 3) Cell-lineage level: This level aggregates multiple cell types into broader cell lineages, facilitating the capture of generalized regulatory programs. This multi-scale annotation allows our model to learn shared features across lineages and types while making specific predictions at the cellular level.

To ensure a fair comparison, we kept all Scanpy parameters identical across the Baseline, MTL_Lineage, and MTL_Celltype models. The input for the clustering analysis was the predicted expression matrix, restricted to a consistent set of 549 Highly Variable Genes (HVGs). The exact parameter settings used are as follows: 1) HVG selection: Selection was performed using the standard Scanpy workflow (sc.pp.highly_variable_genes) without specifying a fixed number of top genes (n_top_genes=None) and with default thresholds (min_mean=0.0125, max_mean=3, min_disp=0.5). 2) Neighborhood Graph: sc.pp.neighbors was run with n_neighbors=15 and use_rep='X_pca' (based on the top 50 PCs). 3) Louvain Clustering: sc.tl.louvain was performed with a fixed resolution=1.0 to identify cell clusters. 4) t-SNE Visualization: sc.tl.tsne was executed with a fixed random_state=42 to ensure the reproducibility of the layouts.

### **12 Prediction performance of MTL framework**

To evaluate the predicted cell relationship predicted from the held-out chr8, the predicted gene expression of single cell was projected into a two-dimensional embedding using *t*-SNE, following the standard procedure in Scanpy procedure, to establish a baseline model. We then quantitatively assessed the clustering similarity between the predicted cell atlas and the actual atlas using Adjusted mutual information (AMI), Adjusted Rand index (ARI), and Normalized Mutual Information (NMI). These metrics were calculated by comparing the Louvain clusters derived from the predicted data against the ground-truth annotations of Cell-type level and Cell-lineage level. This multi-faceted comparison provides a nuanced assessment of the model's ability to recapitulate underlying biological relationships.

To evaluate the gene expression prediction performance of the CNN model with MTL framework, we calculated the classification metrics, including confusion matrix, Accuracy, Precision, Recall, F1-score, and AUROC values. Furthermore, to assess performance granularity, we compared the AUROC scores across different hierarchical levels including Single-cell level, and Cell-type level, and Cell-lineage level.

### **13 Prediction performance on rare cell types of MTL framework**

To evaluate the performance of the CNN model in classifying rare cell types, we utilized the MCA dataset that comprises 98 cell types and a total of 179,344 cells. From this dataset, we first identified a naturally rare population, ‘Muscle_Mouse98’, containing fewer than 100 cells. We compared classification performance metrics (e.g., confusion matrix, Accuracy, Precision, F1 score, and AUROC) for this rare type against those of common cell types.

To assess the biological relevance of the predictions for ‘Muscle_Mouse98’, we further calculated the Cluster Coherence Ratio to quantify the concordance between a biological cell-type label and the model’s learned latent embeddings through unsupervised clustering, which follows a three-step process: 1) Unsupervised clustering. We perform Louvain clustering on the low-dimensional cell embeddings learned by our model. This process is independently of any known cell-type labels. 2) Distribution analysis. For a specific target cell type, we examine the distribution of its cells across the resulting Louvain clusters. 3) Cluster Coherence Ratio calculation. the ratio is defined as the proportion of cells from a given cell type that fall into its largest Louvain cluster.

$$Ratio= \frac{\max\left( N\_ⅈ \right)}{N\_total}$$

where N_i is the number of cells of the type in Louvain cluster i, and N_total is the total number of cells of that type.

A ratio of 1.0 indicates that all 83 cells of Muscle_Mouse98 were assigned to the same unsupervised cluster, demonstrating that the model learned a highly distinct and coherent representation for this population.

To systematically evaluate the robustness, we performed stratified down-sampling on the MCA dataset to generate a subset of 10,000 cells. This process 62 artificially rare populations, each with fewer than 100 cells, allowing for a large-scale assessment of performance on underrepresented classes.

### **14 Biological interpretation**

In the MCA dataset, to systematically identify potential expression signals that may be obscured by technical limitations, we screened a specific gene set through the following pipeline: First, the original scRNA-seq data and the model-generated predicted expression matrices were binarized separately based on their respective median expression values for each gene. Specifically, for each dataset, genes with an expression level higher than or equal to the median were assigned a value of 1 (denoting "expressed"), while those below the median were assigned a value of 0 (denoting "not expressed"). Subsequently, we filtered for genes that were not detected as expressed in the experimental sequencing data (test_target = 0) but were predicted to be expressed by the model (pred_target = 1), ultimately yielding 619 candidate genes. Ultimately, we performed Gene Ontology (GO) enrichment analysis for these genes using the enrichGO function in the R package clusterProfiler (v4.14.6).

### **15 Statistical analyses**

All statistical analysis was performed using R (v.4.3.1) and Python (v.3.8.12). For Figure 1A and S1, per-cell AUROC and PCC values were compared between raw baseline and MAGIC-imputed conditions using the two-sided Wilcoxon signed-rank test, paired by cell (same cell barcode). Sample sizes per platform are provided in Table S1. P-values were adjusted using the Benjamini-Hochberg FDR method within each dataset (Supplementary Table S3). For Figure 1B, Pearson correlation (two-sided) was computed between mean detected-gene count and mean PCC across platforms. For Figure 1C, 1E, and S3, pairwise Wilcoxon rank-sum tests were performed within each hyperparameter category; p-values were adjusted using the BH-FDR method within each category. For Figure 1G–H, architecture performance was compared using Wilcoxon rank-sum test across n = 5 independent training runs per architecture; p-values were Bonferroni-corrected (α = 0.01). Boxplots display median, IQR, and 1.5× IQR whiskers. Significance thresholds: * p < 0.05, ** p < 0.01, *** p < 0.001, **** p < 0.0001.

## **Reference**

Han, X., Wang, R., Zhou, Y., Fei, L., Sun, H., Lai, S., Saadatpour, A., Zhou, Z., Chen, H., Ye, F.*, et al.* (2018). Mapping the Mouse Cell Atlas by Microwell-Seq. Cell *172*, 1091-1107.e1017.

Han, X., Wu, H., Wang, X., Liu, D., Fu, Y., Yang, L., Wang, R., Zhang, P., Wang, J., Ma, L.*, et al.* (2025). Modeling the vertebrate regulatory sequence landscape by UUATAC-seq and deep learning. Cell.

Hashimshony, T., Senderovich, N., Avital, G., Klochendler, A., de Leeuw, Y., Anavy, L., Gennert, D., Li, S., Livak, K. J., Rozenblatt-Rosen, O.*, et al.* (2016). CEL-Seq2: sensitive highly-multiplexed single-cell RNA-Seq. Genome Biology *17*, 77.

Jaitin, D. A., Kenigsberg E Fau - Keren-Shaul, H., Keren-Shaul H Fau - Elefant, N., Elefant N Fau - Paul, F., Paul F Fau - Zaretsky, I., Zaretsky I Fau - Mildner, A., Mildner A Fau - Cohen, N., Cohen N Fau - Jung, S., Jung S Fau - Tanay, A., Tanay A Fau - Amit, I., and Amit, I. (2014). Massively parallel single-cell RNA-seq for marker-free decomposition of tissues into cell types. Science *14*, 775-779.

Macosko, Evan Z., Basu, A., Satija, R., Nemesh, J., Shekhar, K., Goldman, M., Tirosh, I., Bialas, Allison R., Kamitaki, N., Martersteck, Emily M.*, et al.* (2015). Highly Parallel Genome-wide Expression Profiling of Individual Cells Using Nanoliter Droplets. Cell *161*, 1202-1214.

Picelli, S., Faridani, O. R., Björklund, Å. K., Winberg, G., Sagasser, S., and Sandberg, R. (2014). Full-length RNA-seq from single cells using Smart-seq2. Nature Protocols *9*, 171-181.

Ramsköld, D., Luo, S., Wang, Y.-C., Li, R., Deng, Q., Faridani, O. R., Daniels, G. A., Khrebtukova, I., Loring, J. F., Laurent, L. C.*, et al.* (2012). Full-length mRNA-Seq from single-cell levels of RNA and individual circulating tumor cells. Nature Biotechnology *30*, 777-782.

Soumillon, M., Cacchiarelli, D., Semrau, S., van Oudenaarden, A., and Mikkelsen, T. S. (2014). Characterization of directed differentiation by high-throughput single-cell RNA-Seq. bioRxiv, 003236.

van Dijk, D., Sharma, R., Nainys, J., Yim, K., Kathail, P., Carr, A. J., Burdziak, C., Moon, K. R., Chaffer, C. L., Pattabiraman, D.*, et al.* (2018). Recovering Gene Interactions from Single-Cell Data Using Data Diffusion. Cell *174*, 716-729.e727.

Wang, X., He, Y., Zhang, Q., Ren, X., and Zhang, Z. Direct Comparative Analyses of 10X Genomics Chromium and Smart-seq2.

Wolf, F. A., Angerer, P., and Theis, F. J. (2018). SCANPY: large-scale single-cell gene expression data analysis. Genome Biology *19*, 15.

Ziegenhain, C., Vieth, B., Parekh, S., Reinius, B., Guillaumet-Adkins, A., Smets, M., Leonhardt, H., Heyn, H., Hellmann, I., and Enard, W. (2017). Comparative Analysis of Single-Cell RNA Sequencing Methods. Molecular Cell *65*, 631-643.e634.
